# Supplementary material for: Diminished measles immunity after paediatric liver transplantation—A retrospective, single-centre, cross-sectional analysis
Source: PLoS One. 2024 Feb 5;19(2):e0296653. doi: 10.1371/journal.pone.0296653 (PMC10843477; doi:10.1371/journal.pone.0296653)
Supplement: S1 Table — (PDF) [file pone.0296653.s001.pdf]

Characteristic	Biliary/cholestatic disease							Malignant liver disease							Acute liver failure							Metabolic disease							Cryptogenic cirrhosis/others						
	**Univariable Cox Regression**				**Multivariable Cox Regression**			**Univariable Cox Regression**				**Multivariable Cox Regression**			**Univariable Cox Regression**				**Multivariable Cox Regression**			**Univariable Cox Regression**				**Multivariable Cox Regression**			**Univariable Cox Regression**				**Multivariable Cox Regression**		
**Characteristic**	**N**	**HR**	**95% CI**	**p-value**	**HR**	**95% CI**	**p-value**	**N**	**HR**	**95% CI**	**p-value**	**HR**	**95% CI**	**p-value**	**N**	**HR**	**95% CI**	**p-value**	**HR**	**95% CI**	**p-value**	**N**	**HR**	**95% CI**	**p-value**	**HR**	**95% CI**	**p-value**	**N**	**HR**	**95% CI**	**p-value**	**HR**	**95% CI**	**p-value**
Sex																																			
Female	26	—	—					6	—	—		—	—		8	—	—		—	—		13	—	—					4	—	—				
Male	43	0.80	0.45, 1.41	0.434				10	0.26	0.05, 1.34	**0.108**	53.5	0.23, 12,195	0.15	5	0.28	0.05, 1.43	**0.125**	9.12	0.01, 8,874	0.53	7	0.61	0.22, 1.68	0.344				3	0.53	0.09, 3.24	0.493			
Living related donation																																			
No	56	—	—		—	—		16	—	—					13	—	—					18	—	—					5	—	—				
Yes	13	0.47	0.20, 1.09	**0.080**	0.26	0.10, 0.74	**0.011**	0							0							2	1.17	0.26, 5.30	0.834				2	0.51	0.05, 4.74	0.553			
Type of graft																																			
Split graft	45	—	—					10	—	—					11	—	—					9	—	—					6	—	—				
Whole graft	24	1.29	0.74, 2.23	0.363				6	0.40	0.09, 1.78	0.231				2	0.65	0.12, 3.68	0.628				11	0.97	0.37, 2.55	0.958				1	1.94	0.17, 21.7	0.589			
Age at transplantation in years	69	0.96	0.87, 1.06	0.407				16	0.96	0.77, 1.20	0.724				13	0.91	0.75, 1.10	0.324				20	0.85	0.75, 0.98	**0.022**	1.01	0.84, 1.22	0.93	7	1.02	0.76, 1.38	0.897			
Vaccinations prior to transplantation																																			
1 vaccine dose	21	—	—					7	—	—		—	—		3	—	—					0	—	—					0	—	—				
2 vaccines doses	48	1.37	0.72, 2.59	0.334				9	6.70	0.82, 55.0	**0.077**	288	0.17, 476,927	0.13	10	1.00	0.11, 9.00	0.999				20							7						
Age at 1st vaccination in years	69	1.26	1.01, 1.57	**0.039**	6.81	3.80, 12.2	**<0.001**	16	0.25	0.02, 3.60	0.310				13	0.00	0.00, 607	0.363				20	1.14	0.89, 1.45	0.299				7	0.00	0.00, 4,280	0.363			
Age at 1st vaccination below 1 year																																			
No	35	—	—					11	—	—					5	—	—					11	—	—					5	—	—				
Yes	34	0.90	0.52, 1.56	0.705				5	1.89	0.34, 10.4	0.466				8	1.83	0.42, 8.03	0.422				9	1.33	0.49, 3.58	0.576				2	1.94	0.17, 21.7	0.589			
Seroprevalence at time of transplantation																																			
No	18	—	—		—	—		0	—	—					0	—	—					2	—	—					3	—	—				
Yes	51	2.18	1.02, 4.65	**0.044**	3.44	1.31, 9.05	**0.012**	16							13							18	—	—	0.998				4	1.97	0.21, 18.3	0.553			
Age at testing in years	69	0.38	0.29, 0.49	**<0.001**	0.14	0.08, 0.25	**<0.001**	16	0.32	0.14, 0.77	**0.011**	0.02	0.00, 2.72	0.12	13	0.05	0.00, 2.29	**0.124**	0.00	0.00, 5,066	0.43	20	0.40	0.25, 0.64	**<0.001**	0.40	0.24, 0.66	**<0.001**	7	—	—	0.994			
Biopsy proven acute rejection (RAI-Score≥3)																																			
No	46	—	—					9	—	—					5	—	—					13	—	—		—	—		3	—	—				
Yes	23	1.40	0.80, 2.45	0.240				7	1.06	0.21, 5.44	0.941				8	0.93	0.23, 3.80	0.924				7	0.36	0.12, 1.07	**0.067**	0.88	0.22, 3.51	0.85	4	1.22	0.20, 7.39	0.832			
Immunosuppression																																			
Cyclosporine A	8	—	—		—	—		5	—	—					1	—	—					0	—	—					0	—	—				

Characteristic	Biliary/cholestatic disease							Malignant liver disease							Acute liver failure							Metabolic disease							Cryptogenic cirrhosis/others						
	**Univariable Cox Regression**				**Multivariable Cox Regression**			**Univariable Cox Regression**				**Multivariable Cox Regression**			**Univariable Cox Regression**				**Multivariable Cox Regression**			**Univariable Cox Regression**				**Multivariable Cox Regression**			**Univariable Cox Regression**				**Multivariable Cox Regression**		
**Characteristic**	**N**	**HR**	**95% CI**	**p-value**	**HR**	**95% CI**	**p-value**	**N**	**HR**	**95% CI**	**p-value**	**HR**	**95% CI**	**p-value**	**N**	**HR**	**95% CI**	**p-value**	**HR**	**95% CI**	**p-value**	**N**	**HR**	**95% CI**	**p-value**	**HR**	**95% CI**	**p-value**	**N**	**HR**	**95% CI**	**p-value**	**HR**	**95% CI**	**p-value**
Tacrolimus	61	2.84	1.01, 7.93	**0.047**	2.62	0.77, 8.92	0.12	11	0.84	0.20, 3.57	0.817				12	0.41	0.05, 3.65	0.422				20							7						
Intensified immunosuppression																																			
No	56	—	—		—	—		15	—	—					11	—	—					13	—	—					6	—	—				
Yes	13	2.97	1.50, 5.90	**0.002**	1.78	0.59, 5.41	0.31	1	3.30	0.34, 32.0	0.303				2	1.21	0.22, 6.65	0.827				7	0.90	0.33, 2.45	0.841				1	1.07	0.11, 10.6	0.953			
